# High incidence and viral load of HHV-6A in a multi-centre kidney transplant cohort

**DOI:** 10.3389/frtra.2023.1188535

**Published:** 2023-06-26

**Authors:** Arturo Blazquez-Navarro, Toralf Roch, Patrizia Wehler, Sviatlana Kaliszczyk, Chris Bauer, Constantin Thieme, Kamil S. Rosiewicz, Ulrik Stervbo, Moritz Anft, Petra Reinke, Christian Hugo, Panagiota Zgoura, Richard Viebahn, Timm Westhoff, Michal Or-Guil, Nina Babel

**Affiliations:** ^1^ Berlin Center for Advanced Therapies (BeCAT), Charité—Universitätsmedizin Berlin, Berlin, Germany; ^2^ Center for Translational Medicine, Universitätsklinikum der Ruhr-Universität Bochum, Medizinische Klinik I, Herne, Germany; ^3^ MicroDiscovery GmbH, Berlin, Germany; ^4^ Universitätsklinikum Carl Gustav Carus, Medizinische Klinik III—Bereich Nephrologie, Dresden, Germany; ^5^ Chirurgische Klinik, Universitätsklinikum Knappschaftskrankenhaus Bochum, Bochum, Germany; ^6^ Institute of Medical Immunology, Charité—Universitätsmedizin Berlin, Berlin, Germany

**Keywords:** renal tranplantation, BK viraemia, CMV (citomegalovirus), epstein—barr virus, HHV-6A/B, transplant outcome

## Abstract

Human herpesvirus 6 (HHV-6) is a common opportunistic pathogen in kidney transplant recipients. Two distinct species of HHV-6, HHV-6A and HHV-6B, have been identified, of which the latter seems to be dominant. However, it is unclear whether they increase the likelihood of other viral reactivations. We characterized a multi-centre cohort of 93 patients along nine study visits for viral load. We tested for the following viruses: HHV-6A and HHV-6B, the herpesviruses cytomegalovirus (CMV) and Epstein-Barr virus (EBV) and the polyomavirus BK (BKV). We detected HHV-6A viral load in 48 (51.6%) patients, while the incidence of HHV-6B was much lower, being detected in 6 (6.5%) patients. The incidence of HHV-6A was higher than of BKV, CMV and EBV. HHV-6A also demonstrated higher viral loads than the rest of viruses. There was a non-significant trend of association between HHV-6A and HHV-6B as co-infection, whereas no increased incidence of other viruses among patients with HHV-6A reactivation was observed. There was no negative effect of high HHV-6A (>10,000 copies/ml) load on markers of renal graft and hepatic function or blood count twelve months post-transplant. In contrast to previously published data, our results show a clear dominance of HHV-6A in peripheral blood when compared to HHV-6B, with higher incidence and viral load levels. Despite the high HHV-6A loads observed, we did not identify any negative effects on posttransplant outcome.

## Introduction

Human herpesvirus 6 (HHV-6) infects over 90% of the healthy population within the first three years of life and is a common opportunistic pathogen in kidney transplant recipients (1). Two distinct species of HHV-6, HHV-6A and HHV-6B, have been identified (1, 2), although the genome of these two viruses have a 90% similarity (3). HHV-6B has been observed to be dominant in peripheral blood and to reactivate often after solid organ transplantation, while HHV6-A seems to be dominant in the central nervous system (1, 2). HHV-6 primary infection occurs in the early childhood and is usually asymptomatic or mild, with rare systemic complications; most symptomatic infections seem to be caused by HHV-6B (4). An even higher incidence of HHV-6 seropositivity (96.4%) has been observed among adult recipients of solid organ transplants (5). As a consequence of immunosuppression, renal transplant recipients (RTR) experience reactivation in 23%–55% of the cases (1). While most reactivations are asymptomatic, they may be associated with graft dysfunction and hepatic dysfunction (1). However, the frequency of these complications and the role of the HHV-6 subtypes, as well as their association with other viral reactivations, have been insufficiently studied. Similarly, to HHV6, herpesviruses cytomegalovirus (CMV) and Epstein-Barr virus (EBV) and the polyomavirus BK (BKV) infection also occurs during childhood, with an approximate prevalence of 80%, 60%, and 90%, respectively (2–4). Reactivations of BKV, CMV and EBV can result in the appearance with clinically relevant symptoms. Especially individuals with an immune system compromised by dialysis or immunosuppressive drugs, i.e., after a solid organ transplantation, bear a high reactivation risk with serve health consequences (5).

Here, we characterized a multi-centre cohort of 93 patients along nine study visits for viral load in peripheral blood (Supplementary Table S1). We tested for the following viruses: HHV-6A and HHV-6B, CMV, EBV and BKV. Patients were tested pre-transplant and one week, two weeks, one month, two months, three months, six months, nine months and twelve months post-transplant. A total of 696 samples were analysed. For more details on the employed methods, see the Supplementary Methods.

**Figure 1:**
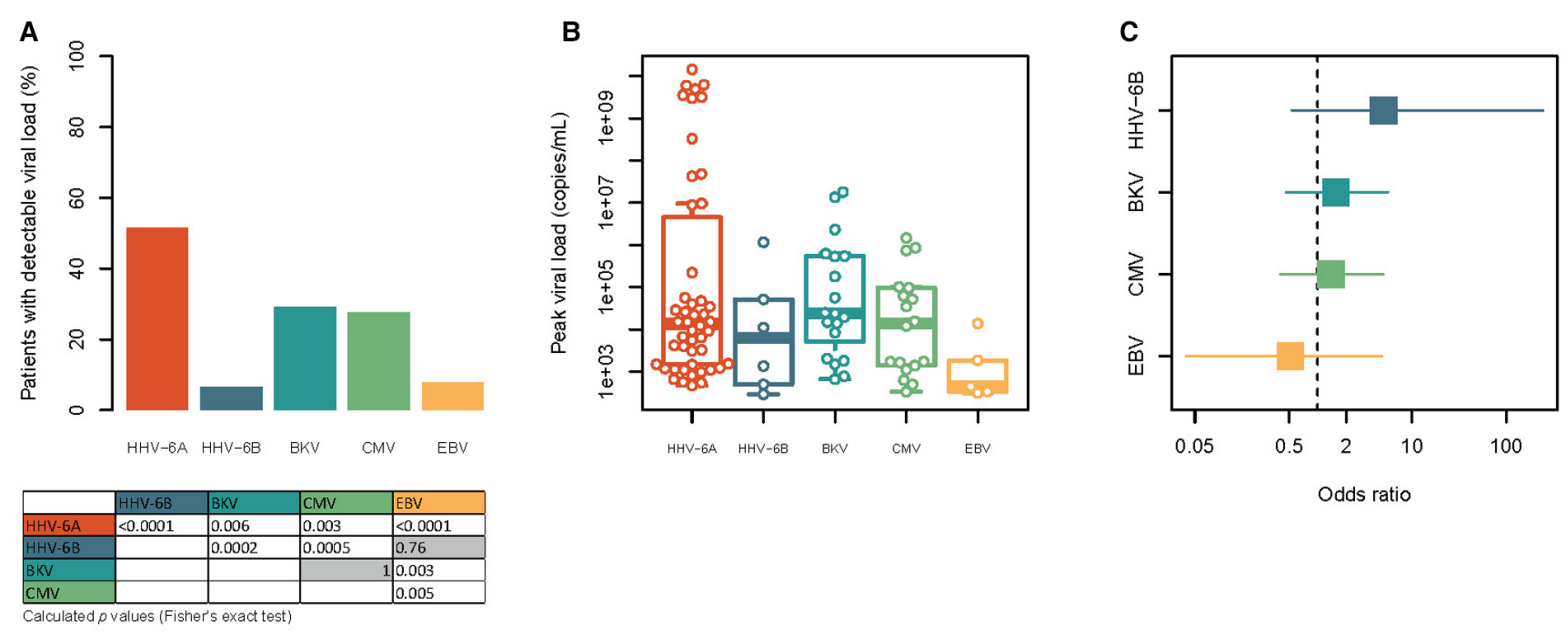
Comparison of the frequency, viral load and mutual association of HHV-6A, HHV-6B, BKV, CMV and EBV in the study cohort. (**A**) Frequency of patients with detectable viremia for HHV-6A, HHV-6B, BKV, CMV and EBV at least one study visit. Statistical significances were tested with Fisher's exact test and are shown in the table below the graph. Non-significant results are shown with gray background. For reasons of clarity only non-redundant comparisons are shown. (**B**) Height of the peak viral load observed for each patient with detectable viraemia for each of the viruses. Note the logarithmic scale. All variables tested by Kruskal-Wallis test with Dunn's post-hoc test showed no significances. (**C**) Forest plot of the association of HHV-6A with the other five viruses. The square points indicate the odds ratio, while the line indicates the 95% confidence interval. The vertical dashed line represents an odds ratio of 1. Note the logarithmic scale.

We detected HHV-6A viral load in 48 (51.6%) patients, while the incidence of HHV-6B was much lower, being detected in 6 (6.5%) patients. The incidence of HHV-6A was higher than of BKV (29.2%), CMV (27.7%) and EBV (7.7%) (Figure 1A). HHV-6A also demonstrated higher viral peak loads [13,600 (1,484–2,378,404) copies/ml] than all other analysed viruses (Figure 1B). Most reactivations occurred for both HHV-6 species pre-transplant or within the first two weeks post-transplant (HHV-6A: 58.3%; HHV-6B: 71.4%). Although not significant there was a clear trend in the association between HHV-6A and HHV-6B [OR: 5.04 (0.53–247.07); *P* = 0.20, Figure 1C], the lack of significance was probably due to the very low incidence of HHV-6B reactivation. The OR of BKV and CMV were similar (1.56 [0.46–5.59] for BKV and 1.38 [0.4–4.97] for CMV). In contrast, the incidence between HHV-6A and EBV showed a reversed associated trend, but also not significant [OR 0.52 (0.04–4.84) Figure 1C]. Lastly, we examined the effects of high viral load of the more common HHV-6A on transplant outcome. Here, we did not find any negative effect of high viral loads (>10,000 copies/ml; *N* = 25) load on markers of renal graft and hepatic function or blood count twelve months post-transplant, when comparing them with patients with no detectable HHV-6A viral load (Supplementary Figure S1).

One limitation of our study is that monitoring the HHV-6 reactivation in the healthy population and among patients with end stage renal disease could not be performed. Thus, it is difficult to judge whether the observed reactivation of HHV-6 in RTR is more prevalent compared to other individuals, especially since we observed a frequent HHV-6 reaction before kidney transplantation. Another aspect of our study is that we did not evaluate specifically chromosomally integrated HHV-6 (ciHHV-6). However, the reported incidence for the healthy population is 0.2% to 2.9% while∼2.0% for renal transplant patients (6, 7), why, this would affect in theory a negligible 3.7% of our HHV-6 positive patient collective. Retrospectively, by calculating the ratio between peak viral load (for HHV-6A) and white blood cell (WBC) counts for every patient we identified just one potentially positive iciHHV-6A positive patient (ratio 1.01) which was not excluded from that study (Supplementary Figure S3). The other patients showed ratios beyond >2.0 or <0.3.

In summary, our results from a multi-centre cohort show a clear dominance of HHV-6A in peripheral blood when compared to HHV-6B and other transplant-associated viruses, with both higher incidence and viral load levels. This cohort are in strong contrast to previously published data (1, 2). We did not identify any significant associations of HHV-6 reactivation with transplant outcome or transplant-associated viral infections such as BKV, CMV and EBV. Moreover, despite the high HHV-6A loads observed we did not identify any negative effects on the graft, nor on hepatic and bone marrow function. Furthermore, it cannot be excluded that other infections also play a role (8). Therefore, the diagnostic utility of HHV-6-PCR should be analysed in larger prospective studies.

## Data Availability

The raw data supporting the conclusions of this article will be made available by the authors, without undue reservation.
