# Supplementary material for: High incidence and viral load of HHV-6A in a multi-centre kidney transplant cohort
Source: Front Transplant. 2023 Jun 26;2:1188535. doi: 10.3389/frtra.2023.1188535 (PMC11235347; doi:10.3389/frtra.2023.1188535)
Supplement: Supplementary file 1 [file Datasheet1.docx]

# Methods

## Patient population and medication

We have conducted the non-interventional, multi-centre, prospective, investigator-initiated e:KID-2 study, which was a validation biomarker substudy of the large prospective randomized trial Harmony (NCT 00724022)^12^. The study was carried out in compliance with the Declaration of Helsinki and Good Clinical Practice and was approved by the local ethics committee of the Charité-Universitätsmedizin Berlin (EA1/112/17). Inclusion criteria were in accordance with the Harmony trial^12^ a renal transplantation from an AB0 compatible donor and with negative crossmatch, panel reactive antibody level lower or equal to 20%, age between 18 and 75 years. Exclusion criteria were a third transplant or a second, if the first was lost due to severe rejection within the first year, combined kidney transplantation with another organ, immunosuppressive therapy up to 6 months before transplantation, HIV positivity, leukopenia and thrombocytopenia ^12^. Women of childbearing age must practice effective contraception.

A total of 93 patients were recruited and monitored along nine study visits. Study visits took place at day 0 (pre-transplant),1st week, 2nd week, 1st month, 2nd month, 3rd month, 6th month, 9th month, and 12th month post-transplant. As this was a non-interventional study, patients were treated according to local protocols.

Patients were treated by a quadruple immunosuppressive therapy including IL-2R antibody as induction therapy, and tacrolimus, MMF and steroids as maintenance therapy. Patients, who did not receive antiviral prophylaxis were monitored for CMV reactivation (pre-emptive approach). The duration of antiviral prophylaxis was 3 months.

## Monitoring of patients

The patient cohort was monitored at the local study centres at the nine pre-defined visits. Thus, estimated glomerular filtration rate (eGFR), as well as full blood count and routine chemistry tests were estimated. eGFR was assessed using the CKD-EPI formula, measured in mL·min^-1^·1.73 m^-2^.^8^

Peripheral blood samples were centrally monitored for HHV-6A, HHV-6B, BKV, CMV, EBV along the nine study visits. To quantify BKV viral DNA copies, DNA was isolated from patient serum using the Ultra Sens virus Kit (Qiagen, Hilden, Germany) according to manufactures instructions and subsequently amplified using the RealStar BKV Kit 1.0 (Altona Diagnostics, Hamburg, Germany) manufactures instructions. For the detection of EBV, CMV, and HHV-6, DNA was isolated from whole blood using the QIAamp DNA Blood Mini Kit (Qiagen). The RealStar EBV Kit 1.0, the RealStar CMV kit 1.0 and the RealStar HHV-6 kit 1.0 (all purchased from Altona Diagnostics) were used according to manufactures instruction to quantify the viral load of EBV, CMV and HHV-6, respectively. The RealStar HHV-6 kit 1.0 allows discrimination and quantification of HHV-6A and HHV-6B specific DNA. As the HHV6-B system can also detect some HHV6-A strains (strong signal for HHV6-A while weak signal for HHV6-B) we classified a HHV6-B signal ct >38 as negative. Supplementary Figure 2 is showing the strong qPCR differences between the HHV-6A and HHV-6B signals intra-patient wise. To stay within an analytical sensitivity rate of 100% detected virus specific DNA for each test system, we defined a concentration of 250 copies ml^-1^. Patients with viral load over detection limit for at least one study visit were defined as positive for the corresponding virus.

## Statistical analysis

Categorical variables are summarised here as numbers and frequencies; quantitative variables are reported as median and interquartile range (IQR). Associations between categorical variables were tested employing the Fisher test and illustrated as a forest plot depicting the odds ratio as square point and 95% confidence interval as a line. The association of outcome variables with HHV-6A reactivation was tested employing the Mann-Whitney U test; here patients with a very high viral load (>10,000 copies·mL^-1^) for at least one visit were compared with patients with no detectable viral load for the value of eGFR, full blood count and chemistry tests one year post-transplant. The cut-off of 10,000 copies·mL^-1^ was used as previously described ^10,11^. A P value below 0.05 was considered significant. GraphPad Prism (Vers. 9.5.1) was also used for statistical analysis and graphical visualization.

## Supplementary data


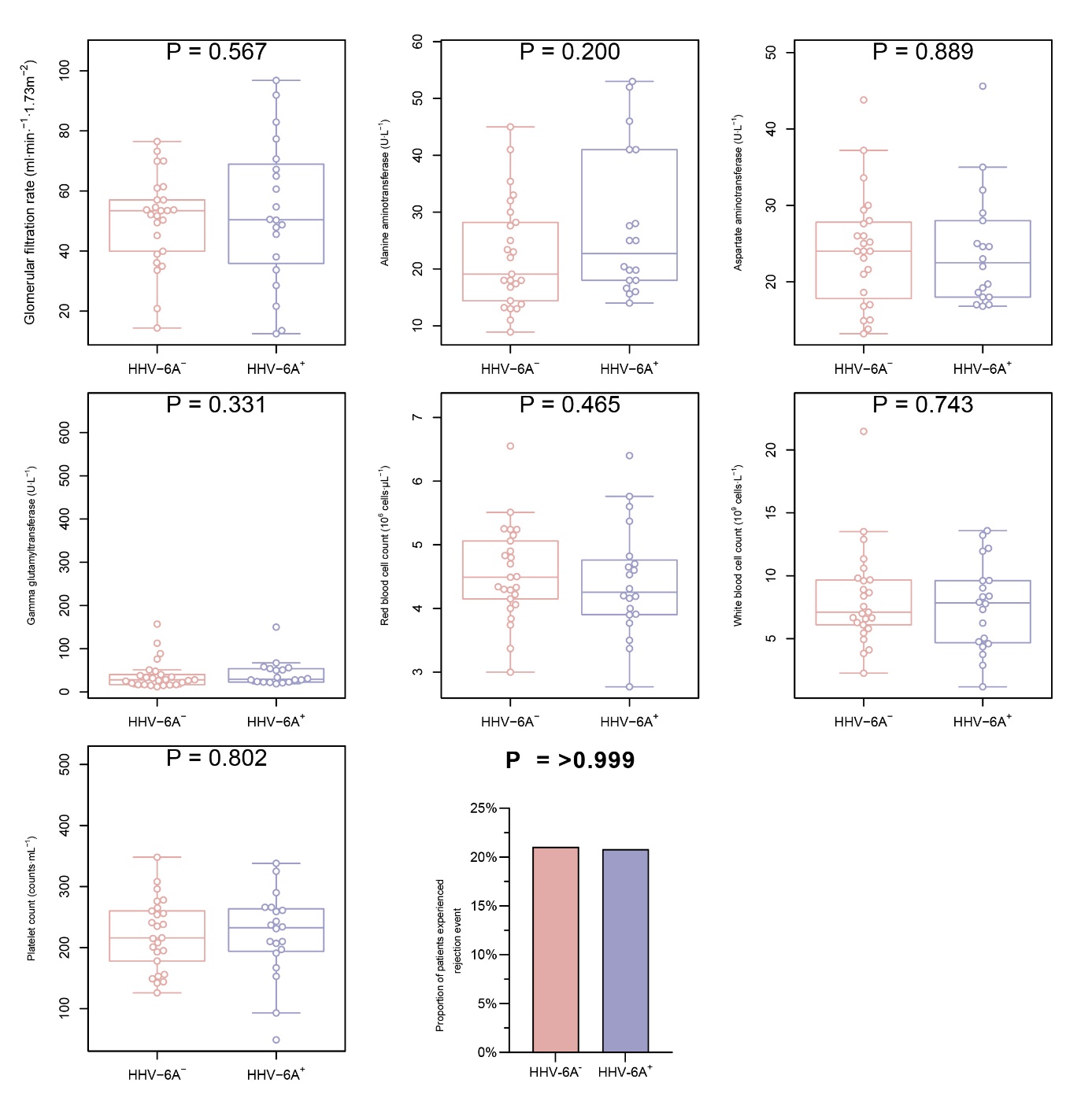


**Figure S1.** **Impact of high HHV-6A viral load during the first post-transplant year on graft renal and hepatic function or blood count twelve months post-transplant.** Patients with a HHV-6A viral load >10,000 copies·mL^-1^ for at least one visit were compared with patients with no detectable HHV-6A viral load during the first post-transplant year. One dot represents one patient at last study visit. The P value was calculated employing the Mann-Whitney U test without adjustment for multiple testing. Proportions of rejection events were tested with Fisher’s exact test.


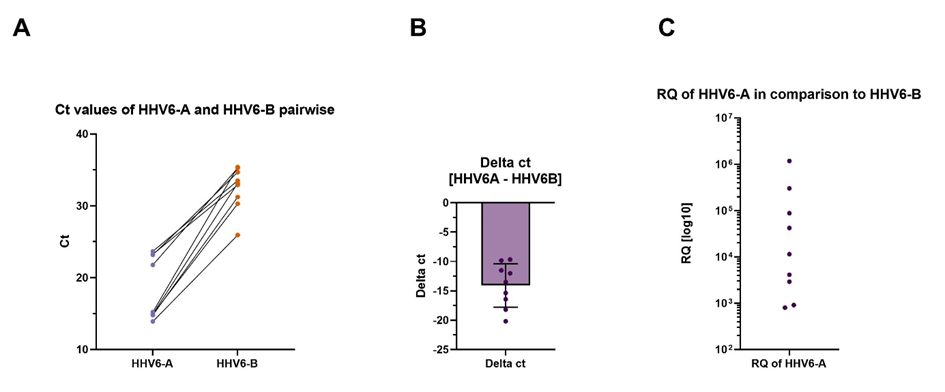


**Figure S2. Comparison of qPCR results of HHV-6A and HHV-6B**. **A** Ct values of HHV-6A and HHV-6B are shown. Results from same patients are connected by a line. **B** Delta ct is plotted (HHV-6A – HHV-6B). **C** Calculated relative quantification (RQ) of HHV-6A in comparison to HHV-6B showed 181,195 times stronger signals (min 803.4; max 1,179,709) for HHV-6A.


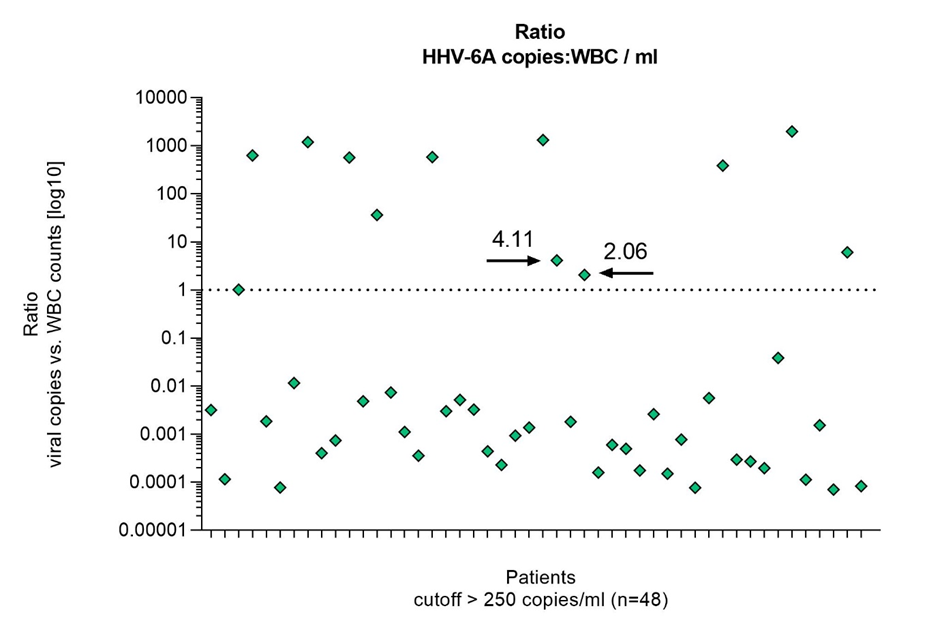


**Figure S3. Result from the calculation of the ratio HHV-6A positive viral copies vs. whited blood cell counts per patient.** Only patients with a peak viral load of >250 copies/ml for HHV-6A were included. The ratio of 1 is indicated by a dashed line.

**Table S1.** **Demographic and treatment characteristics of the patient cohort.**

|  | **Cohort (N=93)** |
| --- | --- |
| **Female patients** | 24 (25.8%) |
| **Age (years)** | 58 [46-66] |
| **Age of donor (years)** | 57 [47-66] |
| **Body mass index (kg·m^-2^)** | 26.1 [23.1-29.7] |
| **Previous transplants** | 6 (6.5%) |
| **Living donation** | 10 (10.8%) |
| **Cold ischaemia time (min)** | 598 [431-782] |
| **CMV high risk constellation*** | 35 (37.6%) |
| **Antiviral valganciclovir prophylaxis** | 47 (50.5%) |
| **Rejection episodes** | 17 (18.3%) |
| **Graft loss** | 0 (0%) |

Categorical variables are shown as numbers and frequencies; quantitative variables are reported as median and interquartile range. *High risk constellation was defined as the combination of a seropositive donor and a seronegative recipient.

## References

1. Lautenschlager, I. & Razonable, R. R. Human herpesvirus-6 infections in kidney, liver, lung, and heart transplantation: Review. *Transpl. Int.* **25**, 493–502 (2012).

2. Ablashi, D. *et al.* Classification of HHV-6A and HHV-6B as distinct viruses. *Arch. Virol.* **159**, 863–870 (2014).

3. Flamand, L., Komaroff, A. L., Arbuckle, J. H., Medveczky, P. G. & Ablashi, D. V. Review, Part 1: Human Herpesvirus-6 – Basic Biology, Diagnostic Testing, and Antiviral Efficac. *Antivir. Ther.* **82**, 1560–1568 (2010).

4. Dewhurst, S., McIntyre, K., Schnabel, K. & Hall, C. B. Human herpesvirus 6 (HHV-6) variant B accounts for the majority of symptomatic primary HHV-6 infections in a population of U.S. infants. *J. Clin. Microbiol.* **31**, 416–418 (1993).

5. Cervera, C. *et al.* A prospective survey of human herpesvirus-6 primary infection in solid organ transplant recipients. *Transplantation* **82**, 979–982 (2006).

6. Aimola, G., Beythien, G., Aswad, A., Kaufer, B. B. Current understanding of human herpesvirus 6 (HHV-6) chromosomal integration. *Antiviral Research* **176**, 104720 (2020).

7. Pellet, P. E., Ablashi, D. V., Ambros, P. F., *et al*. Chromosomally integrated human herpesvirus 6: questions and answers. *Rev. Med. Virol.* **22**, 144-155 (2011).

8. Levey, A. S., Stevens, L. A., Schmid, C. H., *et al*. A New Equation to Estimate Glomerular Filtration Rate. *Ann. Intern. Med.* **150(9)**, 604 – 612 (2009).

9. Tapuchova, I., Pytlik, R., Simara, P., Tesarova, L., Koutna, I. Cytomegalovirus and other herpesviruses after hematopoietic cell and solid organ transplantation: From antiviral drugs to virus-specific T cells. *Transpl. Immunol*. **71**, 101539 (2022)

10. Ogata, M., Satou, T. Kadota, J., et al. Human Herpesvirus 6 (HHV-6) Reactivation and HHV-6 Encephalitis After Allogeneic Hematopoietic Cell Transplantation: A Multicenter, Prospective Study. Clin. Inf. Dis. 57(5), 671-681 (2013).

11. de Koning, C., Admiraal, R., Nierkens, S., Boelens, J. J., Human herpesvirus 6 viremia affects T-cell reconstitution after allogeneic hematopoietic stem cell transplantation. Blood Adv. 2(4), 428-432 (2018).

12. Thomusch O, Wiesener M, Opgenoorth M, Pascher A, Woitas RP, Witzke O, Jaenigen B, Rentsch M, Wolters H, Rath T, Cingöz T, Benck U, Banas B, Hugo C. Rabbit-ATG or basiliximab induction for rapid steroid withdrawal after renal transplantation (Harmony): an open-label, multicentre, randomised controlled trial. *Lancet*. 2016;388:3006-3016. doi:10.1016/S0140-6736(16)32187-0
